# Reverse Takotsubo Cardiomyopathy in the Setting of Acute Asthma Exacerbation

**DOI:** 10.7759/cureus.15469

**Published:** 2021-06-05

**Authors:** Muhammad A Khan, Alexander Howell, Thuy Pham, Nilmarie Guzman

**Affiliations:** 1 Internal Medicine, Orange Park Medical Center, Orange Park, USA; 2 Cardiology, Orange Park Medical Center, Orange Park, USA

**Keywords:** takosubo cardiomyopathy, stress induced cardiomyopathy, asthma exacerbations, short acting beta-agonist, heart disease

## Abstract

Takotsubo cardiomyopathy (TTC) is a reversible form of myocardial injury characterized by transient systolic and diastolic dysfunction secondary to regional left ventricle (LV) wall motion abnormalities. We present a case of a rare variant of TTC, termed reverse TTC (rTTC), involving basal hypokinesis with apical hyperkinesis accounting for less than 5% of identified cases of TTC. Our patient is a 49-year-old Hispanic female who presented for evaluation of dyspnea. She was diagnosed with acute asthma exacerbation. The patient admitted to more frequent use of her albuterol rescue inhaler. Over the course of her hospitalization the patient had elevation of Troponin I and underwent an echocardiogram and coronary angiogram, which revealed the diagnosis of rTTC in the setting of inhaled beta agonist overuse for acute asthma exacerbation. Our case highlights the importance of adequately managing asthma to prevent exacerbation and overuse of inhaled sympathomimetic agents in an effort to decrease the incidence of TTC in the asthmatic population.

## Introduction

We present a case of reverse takotsubo cardiomyopathy (rTTC) in the setting of catecholamine-induced cardiotoxicity secondary to overuse of inhaled short-acting beta agonists (SABA) in acute asthma exacerbation. Takotsubo cardiomyopathy (TTC), also known as broken heart syndrome and stress cardiomyopathy, is a form of reversible myocardial injury characterized by transient (<21 days) systolic and diastolic dysfunction secondary to regional left ventricle (LV) wall motion abnormalities. In typical TTC this involves apical hypokinesis and basal hyperkinesis, while rTTC is classified by basal hypokinesis and apical hyperkinesis [1]. There are additional variants of TTC documented, including mid-ventricular TTC and fast wandering classical TTC [2].

The prevalence of TTC as a whole is likely under-reported due to misdiagnosis of acute coronary syndrome (ACS) or lack of patient presentation when disease is mild. It is estimated to have a prevalence of 15 to 30 per 100,000 per year in the United States and Europe [3]. TTC is more prevalent with increasing age, as <10% of recorded cases have occurred in individuals <55 years of age. Reverse TTC is a rare variant of TTC and accounts for approximately 2-5% of TTC cases and involves less severe hemodynamic compromise compared to typical TTC [4]. rTTC also involves higher level of Troponin I elevation (13.1 ng/ml v. 1.6 ng/ml; p=0.001) and lower levels of B-type natriuretic peptide (BNP) (613.3 pg/ml v. 4987 pg/ml; p=0.020) when compared to typical TTC, further distinguishing it clinically [5].

The pathogenesis of TTC has not been completely elucidated but most hypotheses involve excess central autonomic nervous system stimulation causing excess neurotransmitter release and stimulation on the myocardium. The neurotransmitters with the strongest link to TTC pathogenesis include norepinephrine (NE) and neuropeptide Y (NPY). A similar hypothesis involves catecholamine-induced myocardial stunning [6]. Impaired myocardial perfusion is also observed in TTC, however it is unclear if this contributes to the development of TTC or is simply a consequence of the disease. Neuropeptide Y has an important role in the immune system including stimulation of immune cell migration, T helper cell cytokine release and antibody production. Upregulation of NPY signaling is thought to contribute to the development of many autoimmune and inflammatory diseases, including asthma [7]. Asthma is a known risk factor for the development of TTC and this is likely due to either the upregulation of NPY signaling in asthmatic individuals, the use of sympathomimetic medications to manage asthma, or both.

## Case presentation

A 49-year-old Hispanic female with past medical history of asthma presented to the Emergency Department (ED) with a chief complaint of progressively worsening dyspnea over the past one week. The patient endorsed no alleviation with a rescue albuterol inhaler. She was a non-smoker with occasional alcohol use and no recreational drug use. Family history was significant for hypertension and hyperlipidemia. Her initial vital signs were as follows: heart rate 82/min, blood pressure 135/84 mmHg, 96% on room air, and afebrile. Physical examination was significant for diffuse inspiratory and expiratory wheezing. Cardiovascular examination revealed S1/S2 heart sounds with no murmur gallop or rub. The patient had no jugular vein distention (JVD), peripheral pulses were intact and no cyanosis, clubbing or edema was noted. Initial imaging including a chest X-ray and CT angiogram chest was negative for acute abnormality.

In the emergency department, the patient received nebulization with albuterol and intravenous steroids resulting in alleviation of symptoms. The patient was admitted to the medical floor for further management of acute asthma exacerbation. Over the course of hospitalization, patient’s breathing gradually worsened to the point of requiring non-invasive positive pressure ventilation with bi-level positive airway pressure (BIPAP) as the arterial blood gas revealed acute respiratory acidosis.

**Figure 1:**
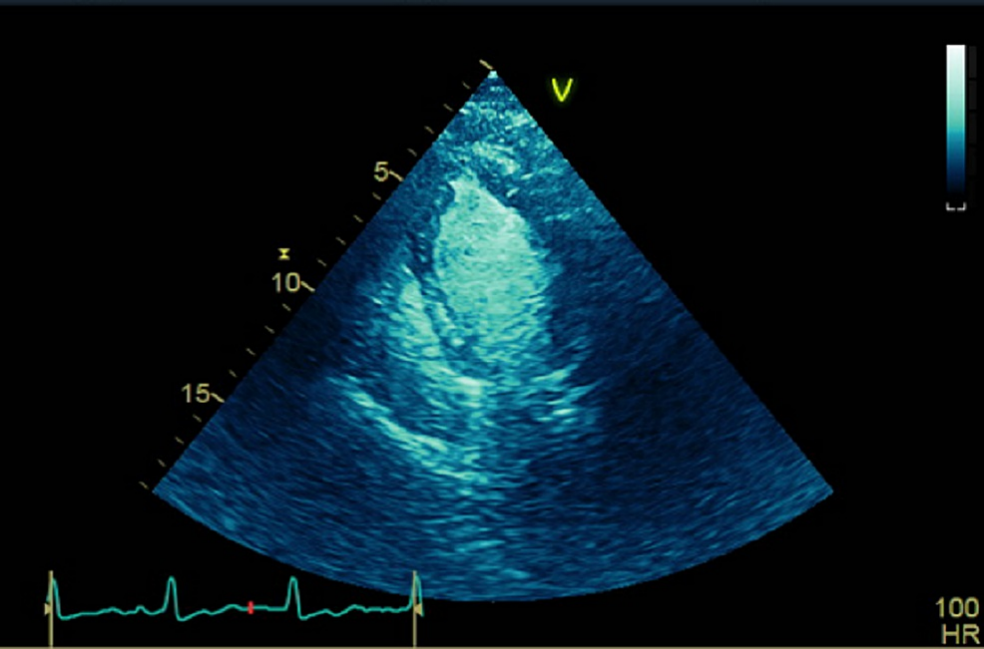
Transthoracic echocardiogram: end systolic, showing contracting left ventricle (LV) apex and mildly dilated base.

**Figure 2:**
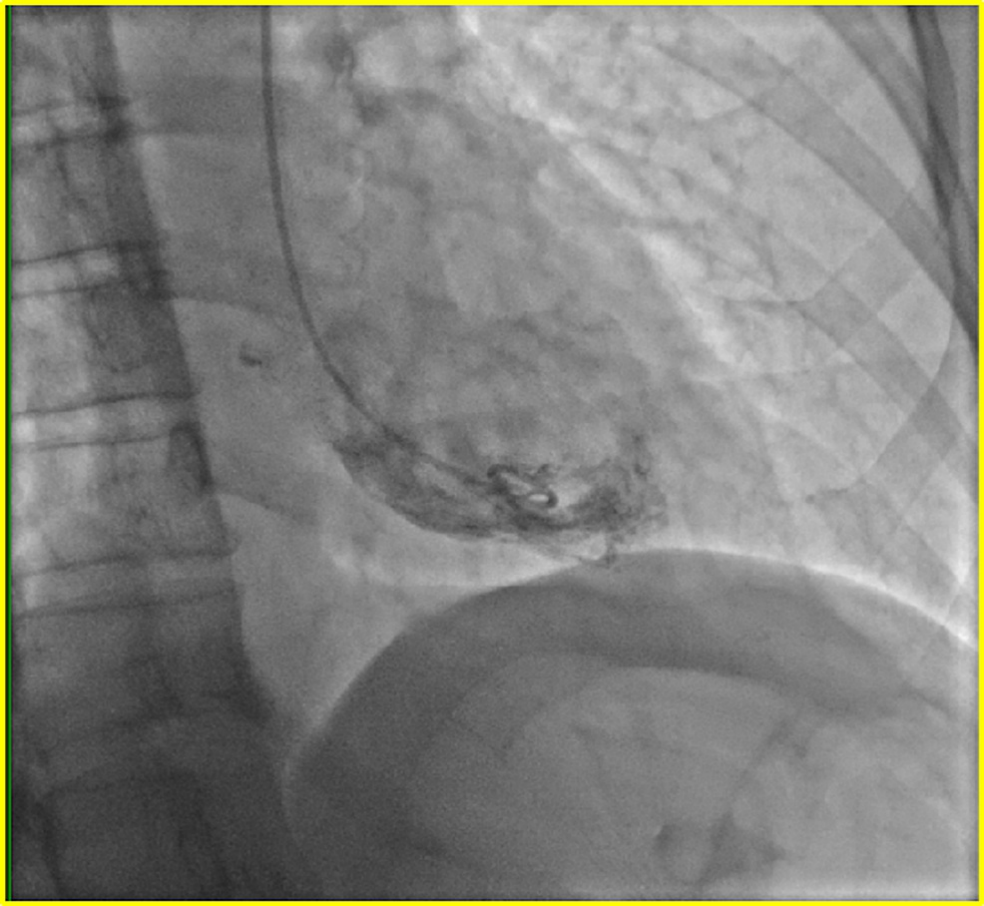
Left ventriculogram showing contracting apex of the heart and mildly dilated base.

The patient was adequately treated with BIPAP resulting in normalization of her arterial blood gases (ABGs) as well as her clinical respiratory status, but her Troponin I continued to trend up to 0.48 ng/ml two hours after her admission, then 2.22 ng/ml eight hours after admission and finally 4.08 ng/ml 11 hours after her admission. An urgent transthoracic echocardiogram (Figure 1) was performed, which revealed: ejection fraction in the range of 40-45%, severe posterior wall and anteroseptal hypokinesis with normal apical function. Cardiology was consulted and patient was taken for an emergent left heart catheterization. The coronary angiography (Figure 2) revealed patent coronary arteries with ejection fraction in the range of 40-45% and proximal anterior and inferior hypokinesis consistent with rTTC. 

Over the course of her hospitalization, her respiratory symptoms improved, she no longer required BIPAP and was stable for discharge three days after her admission. The patient was discharged with instructions to follow up outpatient with cardiology and pulmonology and was given prescriptions for inhaled corticosteroids as well as short-term systemic corticosteroids.

## Discussion

Our patient’s case of rTTC was secondary to the overuse of inhaled short-acting beta agonists as she had no other risk factors and no identifiable stressful event other than her asthma exacerbation to incite her disease process. A case similar to ours has been documented in the literature, however it was classic TTC with apical hypokinesis and was secondary to overuse of inhaled long-acting beta agonists (LABA) [8]. Additionally, there is a report of a 43-year-old female that presented in status asthmaticus that was later diagnosed with classical TTC [9].

A Japanese retrospective study with a sample size of 328 patients determined that patients with rTTC had increased levels of plasma epinephrine in comparison to the patients with classical TTC, revealing that catecholamine-induced cardiotoxicity is likely involved in rTTC pathogenesis. The use of amphetamine and methamphetamine has also been recognized as a trigger for rTTC [10]. This adds to the evidence that sympathomimetic overuse in the form of inhaled albuterol was the inciting event for the development of rTTC in our patient.

Our case in addition to the literature reviewed and mentioned above indicates the need to adequately treat asthma for prevention of TTC, a likely under-diagnosed condition associated with asthma. These diseases are related in their pathogenesis as both involve upregulation of NPY. They are also related in that the use of sympathomimetics for the treatment of asthma exacerbation can be the inciting event for development of TTC. By adequately treating asthma and educating patients in the outpatient setting we could decrease the incidence of exacerbations and thus the need for administration of large quantities of sympathomimetics in these individuals. As a result, this would decrease the incidence of TTC within the asthmatic population.

## Conclusions

Our patient is a 49-year-old Hispanic female who presented for evaluation of dyspnea. She was diagnosed with acute asthma exacerbation requiring non-invasive positive pressure ventilation with BIPAP. During her initial history, the patient admitted to more frequent use of her albuterol rescue inhaler prior to seeking medical care. Over the course of her hospitalization the patient underwent an echocardiogram and coronary angiogram, which revealed the diagnosis of rTTC in the setting of inhaled beta agonist overuse for acute asthma exacerbation. Our case highlights the importance of adequately managing asthma to prevent exacerbation and overuse of inhaled sympathomimetic agents in an effort to decrease the incidence of TTC in the asthmatic population.
